# Antibiotic resistance profiles and associated factors of *Pseudomonas* Infections among patients admitted to large tertiary care hospital from a developing country

**DOI:** 10.1186/s13756-023-01355-4

**Published:** 2023-12-20

**Authors:** Sara Shbaita, Safaa Abatli, Mamoun W. Sweileh, Banan M Aiesh, Ali Sabateen, Husam T. Salameh, Adham AbuTaha, Sa’ed H. Zyoud

**Affiliations:** 1 Department of Medicine, College of Medicine and Health Sciences, An-Najah National University, Nablus, 44839 Palestine; 2 Department of Internal Medicine, An-Najah National University Hospital, Nablus, 44839 Palestine; 3 Infection Control Department, An-Najah National University Hospital, Nablus, 44839 Palestine; 4 Department of Hematology and Oncology, An-Najah National University Hospital, Nablus, 44839 Palestine; 5 Department of Biomedical Sciences, Faculty of Medicine and Health Sciences, An-Najah National University, Nablus, 44839 Palestine; 6 Department of Pathology, An-Najah National University Hospital, Nablus, 44839 Palestine; 7 Department of Clinical and Community Pharmacy, College of Medicine and Health Sciences, An-Najah National University, Nablus, 44839 Palestine; 8 Poison Control and Drug Information Center (PCDIC), College of Medicine and Health Sciences, An-Najah National University, Nablus, 44839 Palestine; 9 Clinical Research Center, An-Najah National University Hospital, Nablus, 44839 Palestine

**Keywords:** *Pseudomonas* aeruginosa, Multidrug-resistant *Pseudomonas*, Antibiotic resistance, MDR

## Abstract

**Background:**

*Pseudomonas* infections are among the most common infections encountered in hospitalized patients, especially those with chronic illnesses or an immunocompromised state. Management of these infections has become challenging due to increased antibiotic resistance. Therefore, this study examines the antibiotic resistance profiles of *Pseudomonas* spp. and the associated factors among patients admitted to a large tertiary hospital in a developing country.

**Methods:**

This retrospective observational chart review study assessed patients admitted to a large tertiary hospital in a developing country with a positive culture growth of *Pseudomonas* from anybody site. Antibiotic susceptibility of the isolated *Pseudomonas* and patient characteristics were studied from the start of 2021 to the end of 2022. The study ground consisted of 185 patients.

**Results:**

The study included 185 patients with positive *Pseudomonas* isolates. Males constituted 54.6% of the sample, while 45.4% were females. The median age of the patients was 53 years. Patient comorbidities and risk factors for *Pseudomonas* infection and multidrug resistance were assessed. Antibiotic resistance to the *Pseudomonas* regimens showed the highest resistance to meropenem and ciprofloxacin (23.4%, similarly) among isolates of *Pseudomonas aeruginosa*. Multidrug resistance (MDR) was found in 108 (58.4%) isolates. The most commonly used antibiotic for treatment was piperacillin-tazobactam, accounting for 33.3% of cases, followed by aminoglycosides at 26.6%.

**Conclusions:**

*Pseudomonas aeruginosa* isolates were resistant to meropenem and ciprofloxacin. Over half of the isolates were multidrug-resistant, which was worrying. Piperacillin-tazobactam and aminoglycosides were the most often utilized antibiotics, highlighting the significance of susceptibility testing. Implementing antimicrobial stewardship programs and infection control measures can help reduce drug resistance and improve outcomes in *Pseudomonas* infections.

## Introduction

*Pseudomonas aeruginosa* is an opportunistic Gram-negative pathogen that can live in various hosts, such as plants, animals, and people [1]. Even in environments with inadequate nutrient levels, it can persist in both community and hospital settings [2, 3]. The prevalence of hospital-acquired infections (HAI) exceeds that of community-acquired infections (CAI) [4, 5]. Patients with community-acquired pneumonia (CAP) in Africa (5.5%), Asia (5.2%), South America (4.9%), North America (4.3%), and Europe (3.8%) are more likely to have *P. aeruginosa* isolates [6]. Numerous nosocomial infections, including bacteremia, urinary tract infections (UTIs), wound infections, and ventilator-associated pneumonia (VAP), are linked to *P. aeruginosa* [7]. It is the fourth most prevalent nosocomial pathogen, with mortality rates among critically ill patients ranging from 27 to 48% [8, 9].

According to the INFORM database, the rates of multidrug-resistant (MDR) *P. aeruginosa* infections in healthcare settings consistently range from 11.5 to 24.7% [10]. The World Health Organization (WHO) has classified carbapenem-resistant *P. aeruginosa* as a priority 1 or “critical” pathogen that requires urgent development of new therapies to address the emerging public health crisis of drug resistance [11]. The Center for Disease Control and Prevention (CDC) has also recognized MDR-*P. aeruginosa* as a significant threat for the past decade, with an estimated 32,600 cases, 2,700 deaths, and healthcare costs amounting to US $767 million annually [12]. A national study in the United States found that patients with MDR-*P. aeruginosa* respiratory infections had higher mortality rates, approximately seven days longer hospital stays, increased readmission rates, and an additional cost of US $20,000 per infection compared to those with non-MDR-*P. aeruginosa* infections [12].

A history of chronic obstructive pulmonary disease (COPD), prolonged hospitalization or intensive care unit (ICU) admission, prior *P. aeruginosa* infection, use of invasive medical devices (such as tracheostomy tubes, urethral catheters), or prior surgery are risk factors for multidrug-resistant *P. aeruginosa* infection [13, 14]. Other common hospital-acquired infections caused by *Pseudomonas* include pneumonia by a ventilator and urinary tract infections by catheters [15]. Individuals with invasive medical devices, such as catheters or endotracheal tubes, are at an increased risk because it can form difficult-to-treat biofilms [16]. There is a 23% [17] chance that patients who contract infections in the ICU will have *P. aeruginosa*, and a 48.7% [18] chance that they will have multidrug-resistant *P. aeruginosa.*

Hospitalized patients who are chronically ill or immunocompromised are at significant risk for *Pseudomonas* infections, especially if they are brought on by strains resistant to antibiotics. The development of *Pseudomonas* strains resistant to antibiotics has made treating these infections even more challenging. In addition to examining the patterns of antibiotic resistance of *Pseudomonas* in patients admitted to a significant tertiary hospital in a developing country, this study will also examine the contributing factors to these patterns. By providing antibiotic resistance profiles, this study will aid clinicians in selecting the most effective antibiotics for *Pseudomonas* infections. This information will improve patient outcomes and reduce *Pseudomonas*-associated mortality rates. Understanding the factors that contribute to the antibiotic resistance of *Pseudomonas* can aid healthcare facilities in implementing targeted infection control measures to prevent the spread of resistant strains and reduce healthcare-associated infections. This study examines *Pseudomonas* infections in a developing country in order to increase our knowledge of antimicrobial resistance and its effect on global healthcare systems. The findings will guide local, national, and international efforts to combat antibiotic resistance and promote prudent antibiotic use. In addition, this study contributes to the epidemiology and management of *Pseudomonas* infections by shedding light on antibiotic resistance in *Pseudomonas* infections in developing countries, thereby filling a research gap and advancing the study of *Pseudomonas* infections. Its findings will inform future research and clinical practices based on evidence.

## Methods

### Study design and setting

The research was a retrospective observational analysis of An-Najah National University Hospital (NNUH) patient charts. The study focused on patients admitted to NNUH who had positive culture growth of *Pseudomonas* spp. from any part of their body. NNUH is a tertiary teaching hospital in Palestine that handles complex cases, surgeries, and procedures from various locations, and it has a total of 127 beds. We studied the epidemiology of *Pseudomonas* pathogens among admitted patients, patients’ clinical characteristics and antibiotic susceptibility of the isolated *Pseudomonas* from the start of January 2021 to December 2022.

### Study population and sample size

The study included all patients who had *Pseudomonas* growth in the inpatient setting from all ages in all hospital departments (surgical, medical, pediatrics, cardiac departments, intensive care units, bone marrow transplant, vascular surgery and emergency department). Both *Pseudomonas* from active surveillance testing and clinical samples were studied. Outpatient samples, including hemodialysis center patients, were excluded because the data were incomplete. Regarding patients with multiple samples, we studied the first nonduplicate from each site and remarked on all sites from where it was isolated and then concluded that the patient had *Pseudomonas* growth from multiple sites as a variable. After excluding 13 patients, data were collected, studied, and analysed for 185 patients during the study period.

### Lab methods

The microbiology lab uses the VITEK® 2 Compact (bioMérieux. Marcy l’Etoile, France) for bacterial identification and antibiotic susceptibility. The VITEK® 2 GN cards were used for identification and VITEK® 2 AST – N222 were used for antibiotic susceptibility. The AST-N222 contains the following antibiotics: amikacin, aztreonam, cefipem, ceftazidime, ciprofloxacin, gentamicin, imipenem, meropenem, minocycline, pefloxacin, piperacillin, piperacillin/tazobactam, rifampicin, ticarcillin, ticarcillin/clavulanic acid, and tobramycin.

### Data collection

Patient data, including demographic and clinical information, were collected from electronic medical records and the hospital’s microbiology system. A standardized data collection sheet was used to record details such as age, sex, comorbidities, history of previous admissions and history of antibiotic use. The sheet also captured information on the department where the culture was obtained, any invasive devices inserted, the timing of bacterial growth onset, and the specific site from which *Pseudomonas* was isolated. Additionally, the study examined the *Pseudomonas* species, the antibiotics used for infection treatment, and the antibiotic sensitivity of the pathogen.

### Ethical considerations

The study protocol, which involved accessing and utilizing patient clinical information, received approval from the *Institutional Review Boards (IRBs) of An-Najah National University*. The data and information were treated as confidential and solely used for clinical research objectives. Patient-specific identifiable information was not disclosed, and numerical codes were used instead of names to ensure privacy.

### Statistical analysis

Data were coded, categorized, and entered into the Social Science Statistical Package (IBM-SPSS), version 21.0. Descriptive statistics were conducted with frequencies and percentages for categorical variables and medians and interquartile ranges (IQRs) for continuous variables. The Pearson test was used to assess the correlations. Either the chi-square or Fisher’s exact test, as appropriate, was used to test the significance between categorical variables. The Mann‒Whitney test was used for differences in the means between categories. The significance level was established at a *p*-value < 0.05.

## Results

### Demographics and clinical profile of the study population

A total of 185 patients met the inclusion criteria for our study. 101 (54.6%) were males, while 84 (45.4%) were females. The median age of the patients was 53 years, with an interquartile range (IQR) of 30 to 66. Regarding comorbidities, 58 (31.4%) patients had solid malignancies, and 34 (18.4%) had hematological malignancies. Additionally, 70 (37.8%) patients had cardiovascular disease, 55 (29.7%) had diabetes mellitus, and 28 (15.1%) had renal abnormalities. Among the population, 13 (7.0%) patients were undergoing dialysis, as shown in Table 1. Approximately 36.2% of the patients were admitted to our hospital due to various infectious causes, while 14.1% were found to be neutropenic at the time of admission. Additionally, 3.2% of the patients were primarily admitted due to COVID-19 infections. In terms of inserted devices, 59 (31.9%) patients had a Foley catheter inserted prior to the isolation of *Pseudomonas*, 49 (26.5%) had a central line, and 36 (19.5%) were intubated for more than 48 h before *Pseudomonas* growth on culture. Among the patients, 41.1% had a previous hospitalization within the last three months, and 29.2% had a history of prior antibiotic use. For further details, please refer to Table 1.

**Table 1:** Demographics and clinical profile of patients with *Pseudomonas* isolates

Variable	n (% of available data)
**Age (years), median and IQR**	53 (30–66)
**Gender**	
Male	101 (54.6)
Female	84 (45.4)
**Diabetes mellitus**	
Yes	55 (29.7)
No	130 (70.3)
**Cardiovascular diseases**	
Yes	70 (37.8)
No	115 (62.2)
**Kidney disease**	
Yes	28 (15.1)
**No** **On dialysis**	157 (84.9)
Yes	13 (7.0)
No	172 (93.0)
**Hematological malignancies**	
Yes	34 (18.4)
No	151 (81.6)
**Solid malignancies**	
Yes	58 (31.4)
No	127 (68.6)
**Cause of admission is infectious**	
Yes	67 (36.2)
No	118 (63.8)
**Coronavirus disease-19**	
Yes	6 (3.2)
No	179 (96.8)
**Previous procedures within the past 3 months**	
Yes	76 (41.1)
No	109 (58.9)
**Intubation**	
Yes	36 (19.5)
No	149 (80.5)
**Central line placement**	
Yes	49 (26.5)
No	136 (73.5)
**Foley’s catheter placement**	
Yes	59 (31.9)
No	126 (68.1)
**Neutropenia at admission**	
Yes	26 (14.1)
No	159 (85.9)
**Previous antibiotics use**	
Yes	54 (29.2)
No	131 (70.8)
**Total**	**185**

### Bacterial isolates

Table 2 displays the distribution of the 185 patients who exhibited *Pseudomonas* growth among different hospital wards. The highest proportion was observed in the internal medicine department, with 39 patients (21.1%), followed by the surgery department, with 37 patients (20.0%), and the surgical intensive care unit, with 22 patients (11.9%). Regarding the onset of *Pseudomonas* acquisitions, most isolates (63.8%) were present on admission, defined as a positive culture obtained within the first three calendar days of admission. Additionally, 29.7% of the patients had the pathogen isolated from multiple body sites. *P. aeruginosa* accounted for 97.8% of the reported *Pseudomonas* species in our patient population, as shown in Table 3. Among the clinical samples (a total of 111 nonduplicate samples) that exhibited *Pseudomonas* growth, urine cultures were the most frequently reported site (36.9%). Regarding active surveillance testing, rectal swabs predominated in detecting *Pseudomonas* growth, accounting for 87.9% of the samples. For more specific information, please refer to Table 4.

**Table 2:** Words where cultures that showed *Pseudomonas* growth were obtained

Ward	N (%)
Surgery	37 (20.0)
Surgical intensive care unit	22 (11.9)
Oncology	11 (5.9)
Internal medicine	39 (21.1)
Vascular surgery	6 (3.2)
Medical intensive care unit	21 (11.3)
Cardiac care unit	11(5.9)
Pediatrics	18 (9.7)
Pediatric intensive care unit	11 (5.9)
Bone marrow transplantation	1 (0.5)
Cardiology	8 (4.3)
**Total**	185

**Table 3:** Infectious profile for the *Pseudomonas* isolated during study design

Variable	n (% of available data)
**Timing**	
Present on admission	118 (63.8)
Hospital acquired infection	67 (36.2) Total 185
**Site**	
Single isolate	78 (70.3)
Multisite isolate	33 (29.7) Total 111
**Multi drug resistance**	
Yes	108 (58.4)
**Species of isolated** ***Pseudomonas***	**N (%)**
*Pseudomonas* aeruginosa	181 (97.8)
*Pseudomonas putida* *Pseudomonas stutzeri*	1 (0.5) 3 (1.6)
**Total**	**185**

**Table 4:** Body sites from where clinical samples and active surveillance testing cultures of *Pseudomonas* were isolated (some were multisite)

Source	N (%)
Urine	41(36.9)
Blood	10 (9.0)
Central venous catheter tip	6 (5.4)
Sputum	62 (23.4)
Wound/Tissue	32 (28.8)
Fluid	11 (9.9)
Others	7(6.3)
**Total**	111 (119.7)*
Rectal	87 (87.9)
Nasal	18 (18.2)
Axillary	2 (2.0)
Groin	4 (4.0)
**Total**	99 (112.1)*

*Overlap presented due to multisite sampling for some patients

### Antibiotic utilization and resistance pattern

Piperacillin-tazobactam was the most commonly prescribed antipseudomonal agent for the treatment of *Pseudomonas* infections, accounting for 33.3% of cases, followed by aminoglycosides at 26.6%. Table 5 provides further details on the utilization of antipseudomonal regimens, with approximately 23.3% of patients receiving combination therapy for the management of *Pseudomonas* infection. Please refer to Table 5 for more comprehensive information on the treatment regimens.

**Table 5:** Antibiotics used to treat *Pseudomonas* infections

Antibiotics	Frequency (%)
Aminoglycosides	24 (26.6)
Colistin	7 (7.8)
Meropenem	11 (12.2)
Ceftazidime	28 (31.1)
Fluoroquinolones (ciprofloxacin or levofloxacin)	21 (23.3)
Piperacillin-tazobactam	30 (33.3)
	Total = 90 (134.3)*
**Antibiotics regimen**	**Frequency (%)**
Single agent	68 (75.6)
Two agents	21 (23.3)
Three agents	1 (1.1)
	**Total = 90 (100)**

*Overlap presented as some patients used more than one antibiotic. Some patients were not treated after culture results, as they either died or were discharged

Regarding the susceptibility of *P. aeruginosa*, the study found resistance rates of 23.4% for meropenem and 22.4% for imipenem. Among the tested agents, *P. aeruginosa* exhibited the highest sensitivity to amikacin. Notably, no cases of resistance to either carbapenem were observed in *P. putida*.

The resistance rates of *P. aeruginosa* isolates to piperacillin-tazobactam and ceftazidime were found to be 20.6% and 18.7%, respectively. Additionally, the resistance rate to cefepime in *P. aeruginosa* was 19.6%. In contrast, *P. putida* exhibited complete resistance to cefepime, ceftazidime, gentamicin, and amikacin. Among the cases of *P. stutzeri*, 33.3% showed resistance to each of these agents. Please refer to Table 6 for a comprehensive overview of the resistance rates of *Pseudomonas* species to different antimicrobial agents.

**Table 6:** Antibiotic resistance pattern of the isolated *Pseudomonas* species

	Meropenem	Imipenem	Piperacillin-tazobactam	Ceftazidime	Amikicin	Gentamicin	Cefepime	Ciprofloxacin
	Resistant isolates (% )							
***Pseudomonas aeruginosa*** (107)*	25 (23.4)	24 (22.4)	22 (20.6)	20 (18.7)	14 (13.1)	23 (21.5)	21 (19.6)	25 (23.4)
***Pseudomonas putida*** **(1)**	0 (00.0)	0 (00.0)	0 00.0)	0 (00.0)	0 (00.0)	0 (00.0)	0 (00.0)	0 (00.0)
***Pseudomonas stutzeri*** **(3)**	0 00.0)	1 (33.3)	1 (33.3)	1 (33.3)	1 (33.3)	1 (33.3)	1 (33.3)	0 (00.0)

^a^The bold values indicate *p* < 0.05

^b^Statistical significance values calculated using the Pearson chi-square test

### MDR isolates and their correlations with risk factors

Of the 185 patients included in the study, 108 (58.4%) were found to have a multidrug-resistant (MDR) isolate. The presence of MDR showed no statistically significant association with sex, age, or the presence of various comorbidities but was significantly correlated with intubation or ventilation history at the current admission and before obtaining the culture, in which 28 (25.9%) MDR patients were intubated, while among non-MDR patients, only 8 (10.4%) were intubated (*p* = 0.009). Furthermore, neutropenia on admission was significantly associated with MDR isolates as well (*p* = 0.008). For further details, please refer to Table 7. In terms of risk factors for *Pseudomonas* acquisition onset, either as present on admission or hospital-onset, age was of statistical significant difference (*p* = 0.035) between the two groups along with invasive devices inserted; which were all related to hospital onset *Pseudomonas (p* < 0.001) as shown in Table 8.

**Table 7:** Relations between risk factors and multidrug resistance

Variables	Patient with MDR *pseudomonas* (%) n = 108	Patient without MDR *pseudomonas* (%) n=	*P* value^a^
**Gender**			
Male	56 (51.9)	45 (58.4)	0.375 ^b^
Female	52 (48.1)	32 (41.6)	
**Age**	50.5 [17–66]	55.0 [34.5–66]	0.097 ^d^
**Diabetes mellitus**			
Yes	34 (31.5)	21 (27.3)	0.537 ^b^
No	74 (68.5)	56 (72.2)	
**Cardiovascular disorders**			
Yes	42 (38.9)	28 (36.4)	0.727 ^b^
No	66 (61.1)	49 (63.6)	
**Kidney diseases**			
Yes	17 (15.7)	11 (14.3)	0.785 ^b^
No	91 (84.3)	66 (85.7)	
**Liver diseases**			
Yes	8 (7.4)	6 (7.8)	0.922 ^b^
No	100 (92.6)	71 (92.2)	
**Solid malignancies**			
Yes	28 (25.9)	30 (39.0)	0.060 ^b^
No	80 (74.1)	47 (61.0)	
**Hematological malignancies**			
Yes	20 (18.5)	14 (18.2)	0.954 ^b^
No	88 (81.5)	63 (81.8)	
**Infection was the main cause of admission**			
Yes	38 (35.2)	29 (37.7)	0.730 ^b^
No	70 (64.8)	48 (62.3)	
**Coronavirus disease19**			
Yes	5 (4.6)	1 (1.3)	0.403 ^c^
No	103 (95.4)	76 (98.7)	
**Surgeries in the last 3 months**			
Yes	46 (42.6)	30 (39.0)	0.621^b^
No	62 (57.4)	47 (61.0)	
**History of antibiotics use in the last 3 months**			
Yes	79 (73.1)	52 (67.5)	0.408 ^b^
No	29 (26.9)	25 (32.5)	
**Intubation/ventilation**			
Yes	28 (25.9)	8 (10.4)	**0.009** ^**b**^
No	80 (74.1)	69 (89.6)	
**Central line catheter**			
Yes	32 (29.6)	17 (22.1)	0.251^b^
No	76 (70.4)	60 (77.9)	
**Foley catheter**			
Yes	40 (37.0)	19 (25.0)	0.085^b^
No	68 (63.0)	57 (75.0)	
**On dialysis**			
Yes	7 (6.5)	6 (7.8)	0.731 ^b^
No	101 (93.5)	71 (92.2)	
**Neutropenia on admission**			
Yes	9 (8.3)	17 (22.1)	**0.008** ^**b**^
No	99 (91.7)	60 (77.9)	

^a^The bold values indicate *p* < 0.05

^b^Statistical significance values calculated using Pearson chi-square test ^c^Statistical significance values calculated using Fisher’s exact test

^d^Statistical significance values calculated using the Mann‒Whitney test

**Table 8:** Relations between risk factors and *Pseudomonas* acquisition onset

Variables	Hospital-onset *pseudomonas* (%) n = 67	Present on admission *pseudomonas* (%) n = 118	*P*-value^a^
**Gender**			
Male Female	37 (55.2) 30 (44.8%)	64 (54.2) 54 (45.8)	0.897 ^b^
**Age**	42 [24–62]	55 [33-66.25]	**0.035** ^**d**^
**Diabetes mellitus**			
Yes	20 (29.9)	35 (29.7)	0.978 ^b^
No	47 (70.1)	83 (70.3)	
**Cardiovascular disorders**			
Yes	26 (38.8)	44 (37.3)	0.838 ^b^
No	41 (61.2)	74 (62.7)	
**Kidney diseases**			
Yes	10 (14.9)	18 (15.3)	0.952 ^b^
No	57 (85.1)	100 (84.7)	
**Liver diseases**			
Yes	7 (10.4)	7 (5.9)	0.264 ^b^
No	60 (89.6)	111 (94.1)	
**Solid malignancies**			
Yes	18 (26.9)	40 (33.9)	0.322 ^b^
No	49 (73.1)	78 (66.1)	
**Hematological malignancies**			
Yes	9 (13.4)	25 (21.2)	0.191 ^b^
No	58 (86.6)	93 (78.8)	
**Infection was the main cause of admission**			
Yes	19 (28.4)	48 (40.7)	0.094 ^b^
No	48 (71.6)	70 (59.3)	
**Coronavirus disease 19**			
Yes	2 (3.0)	4 (3.4)	0.999 ^c^
No	65 (97.0)	114 (96.6)	
**Surgeries in the last 3 months**			
Yes	32 (47.8)	44 (37.3)	0.164^b^
No	35 (52.2)	74 (62.7)	
**History of antibiotics use in the last 3 months**			
Yes	52 (77.6)	79 (66.9)	0.125 ^b^
No	15 (22.4)	39 (33.1)	
**Intubation/ventilation**			
Yes	29 (43.3)	7 (5.9)	< **0.001**^**b**^
No	38 (56.7)	111 (94.1)	
**Central line catheter**			
Yes	34 (50.7)	15 (12.7)	< **0.001**^**b**^
No	33 (49.3)	103 (87.3)	
**On dialysis**			
**Yes**	7 (10.4)	6 (5.1)	0.170 ^b^
**No**	60 (89.6)	112 (94.9)	
**Foley catheter**			
Yes	37 (55.2)	22 (18.8)	< **0.001**^**b**^
No	30 (44.8)	95 (81.2)	
**Neutropenia on admission**			
Yes	8 (11.9)	18 (15.3)	0.533^b^
No	59 (88.1)	100 (84.7)	
**Multidrug-resistant** ***pseudomonas***			
Yes	42 (62.7)	66 (55.9)	0.370
No	25 (37.7)	52 (44.1)	

^a^ The bold values indicate *p* < 0.05

^b^ Statistical significance values calculated using Pearson chi-square test

^c^ Statistical significance values calculated using Fisher’s exact test

^d^ Statistical significance values calculated using Mann Whitney test

Relations between risk factors and being infected or colonised with *Pseudomonas* were also studied as illustrated in Table 9. The only statistically significant difference was noted with gender; in which females were more prone to colonisation than males (*p* = 0.005).

**Table 9:** Relations between risk factors and *Pseudomonas* colonisation or infection

Variables	Patient with *pseudomonas* colonization (%) n = 67	Patient with *pseudomonas* infections (%) n = 118	*P*-value^a^
**Gender**			
Male	31 (41.9)	70 (63.1)	**0.005** ^**b**^
Female	43 (58.1)	41 (36.9)	
**Age**	49 [14–66]	54 [32–66]	0.163 ^d^
**Diabetes mellitus**			
Yes	21 (28.4)	34 (30.6)	0.743 ^b^
No	53 (71.6)	77 (69.4)	
**Cardiovascular disorders**			
Yes	26 (38.8)	44 (37.3)	0.536 ^b^
No	48 (64.9)	67 (60.4)	
**Kidney diseases**			
Yes	11 (14.9)	17 (15.3)	0.933 ^b^
No	63 (85.1)	94 (84.7)	
**Liver diseases**			
Yes	7 (9.5)	7 (6.3)	0.427 ^b^
No	67 (90.5)	104 (93.7)	
**Solid malignancies**			
Yes	19 (25.7)	39 (35.1)	0.174 ^b^
No	55 (74.3)	72 (64.9)	
**Hematological malignancies**			
Yes	17 (23.0)	17 (15.3)	0.188 ^b^
No	57 (77.0)	94 (84.7)	
**Infection was the main cause of admission**			
Yes	21 (28.4)	46 (41.4)	0.070 ^b^
No	53 (71.6)	65 (58.6)	
**Coronavirus disease 19**			
Yes	3 (4.1)	3 (2.7)	0.611 ^c^
No	71 (95.9)	108 (97.2)	
**Surgeries in the last 3 months**			
Yes	28 (37.8)	48 (43.2)	0.464^b^
No	46 (62.2)	63 (56.8)	
**History of antibiotics use in the last 3 months**			
Yes	52 (70.3)	79 (71.2)	0.895 ^b^
No	22 (29.7)	32 (28.8)	
**Intubation/ventilation**			
Yes	15 (20.3)	21 (18.9)	0.820 ^b^
No	59 (79.7)	90 (81.1)	
**Type of infection**			
HAI	23 (31.3)	44 (39.6)	0.235 ^b^
POA	51 (68.9)	67 (60.4)	
**Central line catheter**			
Yes	17 (23.0)	32 (28.8)	0.377 ^b^
No	57 (77.0)	79 (71.2)	
**On dialysis**			
**Yes**	4 (5.4)	9 (8.1)	0.481 ^c^
**No**	70 (94.6)	102 (91.9)	
**Foley catheter**			
Yes No	22 (29.7) 52 (70.3)	37 (33.6) 73 (66.4)	0.578 ^b^
**Neutropenia on admission**			
Yes	7 (9.5)	19 (17.1)	0.142^b^
No	67 (90.5)	92 (82.9)	

^a^ The bold values indicate *p* < 0.05

^b^ Statistical significance values calculated using Pearson chi-square test

^c^ Statistical significance values calculated using Fisher’s exact test

^d^ Statistical significance values calculated using Mann Whitney test

## Discussion

Given that 97.8% of the isolates in the study were identified as *P. aeruginosa*, this discussion will primarily focus on this specific subtype. *P. aeruginosa* can potentially cause severe and life-threatening infections in patients, particularly due to the global rise in antimicrobial resistance. The increasing resistance of *P. aeruginosa* poses a significant public health threat that requires effective management strategies [19, 20].

The study encompassed a cohort of 185 patients with *Pseudomonas* isolates comprising 101 (54.6%) males and 84 (45.4%) females. The median age of the patients was 53 years, suggesting a higher susceptibility to *Pseudomonas* infections among older adults. Interestingly, our findings align with a previous study conducted in 2022 at a tertiary care center, which also reported a higher proportion of males (51.5%) but with a lower mean age of 37 years among patients with *Pseudomonas* infections [21]. Additionally, a 2019 Zhejiang University survey revealed that men made up 66.2% of the population and that the average age was 58 years [22].

Notably, in 29.7% of the clinical samples, *Pseudomonas* was isolated from multiple sites within the same patient. Among our study participants, most *Pseudomonas* isolates were obtained from urine samples (36.9%), followed by wound cultures (28.8%). These results align with previous studies, where *Pseudomonas* was commonly found in urine samples, indicating a similar prevalence pattern across different research findings [21].

Comorbid illnesses could predispose patients to *Pseudomonas* acquisition. Our study revealed that diabetes mellitus was found in 29.7% of the patients with *Pseudomonas* isolates. However, cardiovascular and renal diseases were found in 37.8% and 15.1% of the population, respectively. Regarding malignancy, solid malignancies affected 31.4% of the patients, while 18.4% of them had hematological malignancies. In accordance with this, a study in Turkey showed that 32% of patients with positive *Pseudomonas* cultures had a chronic illness, and 20% had malignancy [23].

Secondary bacterial infections are a common outcome of viral respiratory tract infections, contributing significantly to increased illness and mortality [24]. Among patients with coronavirus disease 2019 (COVID-19), *P. aeruginosa* was identified as a bacterial respiratory pathogen in 8% of co-infections in the study conducted by Westblade et al. [25]. This finding aligns with the low prevalence of *Pseudomonas* reported in our study involving COVID-19 patients (3.2%). Consequently, these results support the recommendation that routine empirical treatment for *P. aeruginosa* is generally unnecessary unless the patient has a history of infection caused by this organism or suffers from a chronic lung condition associated with *P. aeruginosa* pneumonia, such as bronchiectasis [6].

*P. aeruginosa*, as an opportunistic pathogen, primarily causes infection in patients who have been hospitalized for an extended period of time and have undergone medical applications [23]. Approximately 41% of the patients included in our study had a history of multiple previous hospitalizations, indicating a potential risk factor for *P. aeruginosa* infection. Additionally, approximately 29% of the patients had been exposed to broad-spectrum antibiotics in the last 3 months, further increasing their susceptibility to *P. aeruginosa* infections. Moreover, various physical breaches in host defenses, such as surgical incisions, urinary and vascular catheter insertion, and endotracheal intubation, can compromise the body’s natural barriers and contribute to developing *P. aeruginosa* infections. These factors collectively enhance the likelihood of *P. aeruginosa* acquisition and infection [26]. Our study found that 19.5% of the patients had an endotracheal tube, 26.5% had a central venous catheter and 31.9% were urinary catheterized. In agreement with this, 71% of the patients had invasive intravascular or urinary catheters, and 40% had undergone previous surgery [23].

The location of patients during hospitalization played a crucial role in our study. We observed that *Pseudomonas* infections occurred in 35% of patients admitted to intensive care units (ICUs), including medical, surgical, cardiac, and pediatric units. ICUs are regarded as high-risk units where patients, mostly immunocompromised, will be provided with special interventions, care and monitoring in addition to the advent of invasive procedures and instrumentation. These multiple risk factors increase the likelihood of acquiring an opportunistic infection. This result was similar to a study which found that most of the isolated *Pseudomonas* were from the ICU [27], in addition to an Indian study that showed that the ICU department was the second highest department from which *Pseudomonas* was isolated [28]. *P.aeruginosa* had a prevalence of 14.5%, of which 48.7% were multidrug resistant [29].

Multiple studies investigated the resistance pattern in the matter of antibiotic resistance of *Pseudomonas* organisms. The first was a study in Poland that showed a resistance rate of 67.8% to imipenem, 42.6% to cefepime, and 39.6% to both piperacillin-tazobactam and ciprofloxacin, while an overall lower resistance rates to gentamicin, ceftazidime, amikacin, and meropenem were 37.6%, 33.2%, 30.2%, and 29.2%, respectively [30]. Another study that was carried out in Palestine in 2020 showed that the resistance rate to imipenem was 49%, meropenem 45.1%, ceftazidime 25.5%, and ciprofloxacin 21.6% [31]. Furthermore, a previous study conducted in the haematology department at the same hospital as our study showed 60% resistance to ciprofloxacin, which was the highest among the tested antimicrobial agents, followed by imipenem (59.3%), piperacillin (54.2%), meropenem (48%), and gentamicin (48%). On the other hand, resistance to cefepime and ceftazidime was the lowest (16% and 24%, respectively) [32]. In contrast, our findings in this study revealed the more susceptibility rates of *Pseudomonas* to the tested anti-pseudomonal agents (imipenem and ciprofloxacin 23.4% for each), meropenem (22.4%), gentamicin (21.5%), and piperacillin-tazobactam (20.6%). These results support the findings that the most commonly utilized antipseudomonal agent was piperacillin-tazobactam in 33.3% of the patients who needed treatment, followed by ceftazidime in 31.1%. It is worth mentioning that in our hospital, carbapenems are among the restricted antibiotics; for this, they are only prescribed as per infectious disease team consultation for critically ill patients when other agents cannot be prescribed for any reason, including resistance to those carbapenem-preserving agents.

*P. aeruginosa* is deemed to be multidrug resistant (MDR) if it exhibits resistance to at least one agent in three or more antipseudomonal classes (carbapenems, fluoroquinolones, penicillins, cephalosporins, and aminoglycosides) [33]. Approximately 108 (58.4%) of the *Pseudomonas* isolates in our study were multidrug-resistant (MDR), which is higher than previous national studies. Notably, a study conducted in the Middle East and North Africa region reported a prevalence of 47.6% for MDR *P. aeruginosa*, specifically in Palestine [34]. Worldwide data reported a prevalence of MDR *Pseudomonas* of 55% in Pakistan [35], 47.8% in 2017 in Africa, and 56% in Egypt [35]. The extensive literature review indicates that resistance to *P. aeruginosa* has progressively increased over time in all countries, including Palestine. This rise in resistance can be attributed, in part, to the distinctive characteristics of *P. aeruginosa*. Notably, this bacterium possesses a large genome consisting of 6.3 million base pairs, which is the largest among all known bacteria. [35].

Another possible interpretation for the higher prevalence of MDR *Pseudomonas* isolates in our set up might be due to the increased rate of active surveillance testing according to the in-house screening policy; nevertheless, this does not rule out that the organism has become aggressive due to the overuse of antibiotics, which promotes antibiotic resistance. However, in this study, 70.8% of the patients did not have any documented history of previous use of antibiotics within the last 3 months. In fact, this might be the result of the intensive awareness campaigns and the national action plan for antimicrobial resistance that developed for the years 2020–2024 [36] or the result of the limited access to data on previous admission or antibiotic exposure.

MDR risk factors have raised significant concerns and have been extensively studied. In Colombia, a study investigated suspected risk factors and compared them between susceptible *Pseudomonas* and multidrug-resistant *Pseudomonas* isolates (n = 40). Among these patients, 10% had diabetes mellitus, 11% had renal disease, and 8% had malignancies. The study also revealed that males were more commonly affected by MDR infections, accounting for 51.9% of the cases. These findings align with similar studies, such as research conducted in Brazil, which also reported a higher incidence of MDR infections among males (55.1%) [37].

In the previous three months of admission in a study, 41 patients had received antibiotics. During the same admission or within 48 h before admission, 22 patients had a central line, 26 had a urinary catheter, and 11 had mechanical ventilation [38]. Comparatively, our study found that among patients with MDR infections, 31.5% had diabetes mellitus, 15.7% had kidney diseases, 25.9% had solid malignancies, and 18.5% had hematological malignancies. Among the 108 patients with MDR infections, 42.6% had undergone surgery in the last three months, and 73.1% had used antibiotics in the same time frame. The prevalence of invasive procedures such as central line, urinary catheter, and mechanical ventilation was 29.6%, 37.0%, and 25.9%, respectively).

### Strengths and limitations

This is one of the first studies conducted in Palestine to identify the antibiotic resistance patterns displayed by *Pseudomonas* isolates from patients in tertiary hospitals. In addition, the study aims to determine the prevalence of multidrug-resistant *Pseudomonas* infections in the study population and to examine the utilization of antibiotics for treating *Pseudomonas* infections in a hospital setting. Nevertheless, our study had some limitations. First, relying on retrospective data from medical records introduces the possibility of missing or insufficient data mainly in the aspect of sensitivity pattern of the colonization pathogens. Dependent on the medical records’ quality are the data’s accuracy and completeness. Second, the study was conducted at a single tertiary care hospital in Palestine, which may have limited the findings’ applicability to other settings or populations. In addition, the sample size of 185 patients was relatively small, which may have limited the statistical power required to detect certain associations or patterns. The trial period was limited to two years, with no long-term patient monitoring. As a result, the study lacked the ability to assess the outcomes and long-term consequences of *Pseudomonas* infections.

## Conclusions

In conclusion, this retrospective observational study included patients admitted to a large tertiary hospital in developing countries to examine antibiotic resistance in *Pseudomonas* infections. *Pseudomonas* infections are difficult to treat due to antibiotic resistance, particularly in elderly or immunosuppressed hospitalized patients. The emergence of meropenem and ciprofloxacin resistance in some *Pseudomonas* aeruginosa isolates is particularly concerning. The isolates had a 58.4% prevalence of MDR, highlighting the significance of choosing the best course of action and antibiotics. The most frequently prescribed antipseudomonal medication was piperacillin-tazobactam, followed by aminoglycosides. Larger samples and multicenter studies need to be used to learn more about *Pseudomonas* infections in the developing world. Understanding the effects of multidrug resistance on patient outcomes, mortality rates, and healthcare costs requires longitudinal studies that follow patients over time. Implementing antimicrobial stewardship programs and infection control measures can help reduce drug resistance and improve outcomes in *Pseudomonas* infections.

## Data Availability

Data and materials used in this work are available from the corresponding author upon request.

## Abbreviations

NNUH: An-Najah National University Hospital
*P. aeruginosa*: *Pseudomonas aeruginosa*
*P. maltophilia*: *Pseudomonas maltophilia*
*P. putida*: *Pseudomonas putida*
*P. stutzeri*: *Pseudomonas stutzeri*
*P. putrefaciens*: *Pseudomonas putrefaciens*
ICU: Intensive care unit
AIDS: Acquired immunodeficiency syndrome
MDR: Multidrug-resistant
IRBs: Institutional Review Boards
SPSS: Statistical Package for The Social Sciences
IQR: Interquartile Range
COVID-19: Coronavirus Disease 2019
